# Value of the platelet-to-lymphocyte ratio in the prediction of left ventricular thrombus in anterior ST-elevation myocardial infarction with left ventricular dysfunction

**DOI:** 10.1186/s12872-020-01712-w

**Published:** 2020-09-29

**Authors:** Qian Zhang, Daoyuan Si, Zhongfan Zhang, Chengbing Wang, Haikuo Zheng, Shouping Li, Shijian Huang, Wenqi Zhang

**Affiliations:** 1 Department of Cardiology, China-Japan Union Hospital of Jilin University, Xiantai Street NO.126, Changchun, Jilin China; 2 Department of Neurology, China-Japan Union Hospital of Jilin University, Changchun, Jilin China

**Keywords:** Platelet-to-lymphocyte ratio, Left ventricular thrombus, Anterior ST-segment elevation myocardial infarction, Left ventricular dysfunction, Prediction

## Abstract

**Background:**

The predictors of left ventricular thrombus (LVT) formation are not well defined in the contemporary era, especially in those patients at high risk. We aimed to evaluate whether the platelet/lymphocyte ratio (PLR) is valuable in the determination of LVT formation in patients with anterior ST-elevation myocardial infarction (STEMI) and left ventricular (LV) dysfunction.

**Methods:**

The LVT group (*n* = 46) was identified from anterior STEMI patients with LV dysfunction who were treated with primary percutaneous coronary intervention (PCI) from January 2017 to December 2019 at the China-Japan Union Hospital of Jilin University. The no-LVT group (*n* = 92) were also selected from the same batch of patients and were age- and sex-matched to the patients with LVT. The PLR was determined at admission and was calculated as the ratio of the platelet count to the lymphocyte count using the complete blood count. The presence of LVT was determined by echocardiography.

**Results:**

The PLR were significantly higher in patients with LVT than in no-LVT group (*p* = 0.001). In a receiver operator characteristic curve (ROC) analysis, using a cut-off value of 118.07 (AUC 0.673, 95% CI: 0.574–0.771, *P* = 0.001), the PLR could independently predict the occurrence of LVT. Multivariate analysis showed that an increased PLR (OR = 1.011, 95% CI: 1.004–1.018, *P* = 0.002), the presence of a left ventricular aneurysm (OR = 46.350, 95% CI: 5.659–379.615, *P* < 0.001) and increased DTBT (OR = 1.005, 95% CI: 1.001–1.009, *P* = 0.012) were independent predictors of LVT formation.

**Conclusions:**

In acute anterior STEMI patients with LV dysfunction, an increased PLR and DTBT and the presence of an LV aneurysm were independent predictors of LVT formation. A larger prospective study is warranted to evaluate this result.

**Trial registration:**

This study was registered (May 4, 2019) on Chinese Clinical Trial Registry (ChiCTR-DDD-17011214).

## Background

Left ventricular thrombus (LVT), one of the most common complications of acute anterior myocardial infarction, is linked to the potentially devastating outcome of thromboembolism or stroke [1, 2]. In the era of primary PCI, despite aggressive reperfusion treatment and antithrombotic treatment, the presence of LVT remains high after anterior STEMI [3, 4]. Recently, a prospective multicenter study showed that the incidence of LVT among patients with LV dysfunction after anterior myocardial infarction was up to 26% [5]. Moreover, detection by standard echocardiography may underestimate the incidence, especially in such a high-risk group [6, 7]. The existing biomarkers, however, are not sufficient to predict which of the patients with anterior STEMI is prone to develop LVT, and the identification of additional early predictors would, therefore, be of benefit.

Determining the correlation between clinical and laboratory indicators of thrombosis is the key to the prevention of thromboembolism. The PLR, a new index, provides information about both the inflammatory and thrombosis pathways. Recent evidence [8–10] has shown the meaningful clinical information of the PLR in coronary heart disease and thrombotic diseases (whether diagnostic information or prognostic information). Despite the findings above, data regarding the role of this inflammatory biomarker in LVT are lacking. We conducted a retrospective case-control study to explore the correlation between LVT and the PLR among anterior STEMI patients with LV dysfunction after primary PCI.

## Methods

### Study population and design

**Fig. 1:**
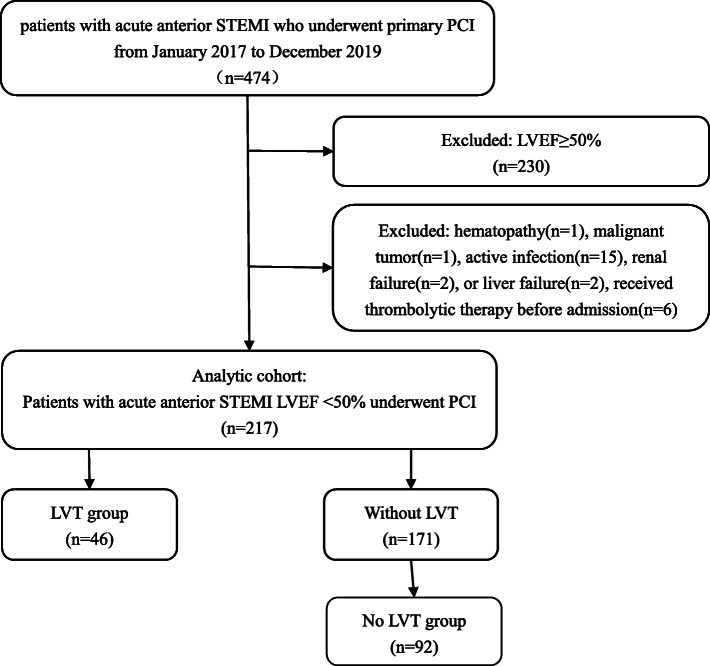
Study Chart Flow. STEMI:ST-Elevation Myocardial Infarction; PCI:percutaneous coronary intervention; LVEF:Left ventricular ejection fraction; LVT:left ventricular thrombus

In this matched case-control study, we screened 474 consecutive patients with acute anterior STEMI who underwent primary PCI from January 2017 to December 2019 at the China-Japan Union Hospital of Jilin University. The exclusion criteria were left ventricular ejection fraction (LVEF) ≥50%, hematopathy, malignant tumor, active infection, renal failure, liver failure, and thrombolytic therapy before admission. In total, 257 patients were excluded due to meeting the following exclusion criteria: LVEF≥50% (*n* = 230), hematopathy (*n* = 1), malignant tumor (*n* = 1), active infection (*n* = 15), renal failure (*n* = 2), liver failure (*n* = 2), and receiving thrombolytic therapy before admission (*n* = 6). The remaining 217 patients with an initial diagnosis of acute anterior STEMI who met the inclusion and exclusion criteria were enrolled. Of them, 46 LVT patients were identified as the LVT group. The no-LVT group (*n* = 92) was matched at a ratio of 1:2 for the variables of age and sex to the patients without LVT during the same period (Fig. 1). The trial was approved by the institutional review board of the China-Japan Union Hospital of Jilin University (Approval No. 2019012804) and was registered in the Chinese Clinical Trial Registry (ChiCTR-DDD-17011214), and written informed consent was obtained from each subject.

Blood samples were taken from the anterior elbow vein on admission before receiving any fibrinolytic therapy. The PLR was calculated as the ratio of the platelet count divided by the absolute lymphocyte count. STEMI was defined according to the WHO criteria as revised in 2000 [11]. The angiographic data of all patients were evaluated by two experienced cardiologists, echocardiography was performed within 7 days after admission, and LVT was defined [12] as a mass with high echo density adjacent to an abnormally contractile myocardial segment. The LVT had to be distinguishable from the surrounding myocardium, have a clear thrombus-blood interface, and be visible in at least two transducer positions. LVEF was calculated by the Simpson method. LV dysfunction was defined as an LVEF< 50%. The door-to-balloon time (DTBT) was recorded as the time from the evaluation by the emergency department (“door” time) to coronary balloon inflation.

### Statistical analysis

The normal distributed continuous variable was expressed by mean ± standard deviation (x bar±SD) and compared using student t-test, and the non-normal distributed continuous variable of was expressed by median (quartile) [M (Q1 ~ Q3)] and compared using Mann-Whitney U test. Chi-square test was used to compare the categorical data. All the independent variables which may affect the dependent variable were performed with univariate analysis. Those variables (PLR, NLR, LV aneurysmn, fibrinogen, DBTB, LVEDD) with statistical significance at the univariate analysis. Multivariate Logistic regression analysis was performed to identify the independent predictors of inflammatory markers to predict the formation of LVT, where the covariates in the multivariate analysis were those statistically significant in univariate analysis (PLR, NLR, LV aneurysmn, fibrinogen, DBTB, LVEDD) with sex and age. The determinants in the analysis was 0.459. The sensitivity and specificity of PLR in predicting LVT were determined by receiver-operating characteristic (ROC) curve analysis, and the diagnostic accuracy was measured by the area under ROC curve (AUROC). All statistical analyses are double-tailed; *p* < 0.05 was considered to be statistically significant. Analyses were conducted using SPSS Version 22.0 ((IBM Corp, Armonk, NY, USA).

## Results

A total of 132 patients participated in this retrospectively matched case-control study. They were divided into the LVT group (*n* = 46) and the no-LVT group (*n* = 92). The main characteristics and hematological features of the patient are shown in Table 1. No significant differences were observed for age, sex, smoking history, or concomitant diseases (such as hypertension and diabetes). Among patients who were admitted to the hospital within 12 h of the onset of chest pain, 17 (37%) developed LVT. In 29 patients, 12 h after the onset of chest pain, their conditions were complicated by LVT. The results showed no significant difference between the two groups (*P* > 0.05). Angiographic analysis showed that 8 patients in the LVT group and 20 patients in the no-LVT group were treated with thrombectomy. The percentages of thrombectomy between the two groups were not significantly different (*P* > 0.05). The average DTBT for patients in the LVT group was 141.87 min, and that for the no-LVT group was 97.58 min. The difference in DTBT between the two groups was statistically significant (*P* < 0.05).

**Table 1:** Baseline Demographics

	Whole cohort(***n*** = 217)	LVT group(***n*** = 46)	No LVT group(***n*** = 92)	95%CI	***P*** value
**Baseline clinical characteristics**					
Age (years)	61.01 ± 11.51	59.63 ± 11.75	61.01 ± 11.51	(0.964,1.030)	0.951
Male sex, n (%)	173 (79.7)	36 (78.3)	173 (79.7)	(0.424,2.359)	1.000
Smoker, n (%)	121 (55.8)	26 (56.5)	121 (55.8)	(0.353,1.497)	0.386
HTN, n (%)	86 (39.6)	15 (32.6)	86 (39.6)	(0.275,1.208)	0.142
DM, n (%)	45 (20.7)	9 (19.6)	45 (20.7)	(0.290,1.547)	0.588
chest pain onset duration<12 h	123 (56.7)	17 (37)	123 (56.7)	(0.260,1.110)	0.091
**Procedural characteristics**					
DTBT (min) (means ± SD)	102.87 ± 91.82	141.87 ± 105.06	102.87 ± 91.82	(1.001,1.008)	0.005
Thrombectomy n (%)	47 (21.7)	8 (17.4)	47 (21.7)	(0.305,1.881)	0.493
Pre-PCI TIMI flowgrade					
0	172 (79.3)	33 (76.1)	172 (79.3)	–	0.155
1	7 (3.2)	1 (2.15)	7 (3.2)	–	0.720
2	8 (3.7)	1 (2.15)	8 (3.7)	–	1.000
3	30 (18.4)	9 (19.6)	30 (18.4)	–	0.144
post-PCI TIMI flowgrade =3					
0	0	0	0	–	_
1	0	0	0	–	_
2	10 (4.6)	1 (2.2)	10 (4.6)	–	1.000
3	207 (95.4)	45 (97.8)	207 (95.4)	–	0.698
**Blood examinations on admission**					
WBC (× 10^9^)	10.05 (8.22, 12.82)	10.7 (7.9, 13.6)	10.05 (8.22, 12.82)	(1.034,1.236)	0.062
MPV (fl)	9.6 (9.0, 10.5)	9.6 (9.1, 10.7)	9.6 (9.0, 10.5)	(0.760,1.415)	0.748
Platelets(×10^9^)	197.5 (158.25, 251.75)	228 (164, 294)	197.5 (158.25, 251.75)	(0.999,1.009)	0.316
Neutrophil(×10^9^)	7.40 (5.60, 10.25)	7.30 (5.45, 10.17)	7.40 (5.60, 10.25)	(1.025,1.234)	0.059
Lymphocyte(×10^9^)	1.68 (1.25, 2.14)	1.41 (1.14, 2.25)	1.68 (1.25, 2.14)	(0.304,0.871)	0.001
Monocyte(×10^9^)	0.63 (0.46, 0.86)	0.58 (0.38, 0.75)	0.63 (0.46, 0.86)	(0.305,5.576)	0.430
NLR	4.38 (2.76, 7.91)	4.37 (2.84, 8.23)	4.38 (2.76, 7.91)	(1.098,1.444)	0.001
PLR	114.57 (90.79, 157.42)	129.6 (98.9194.7)	114.57 (90.79, 157.42)	(1.005,1.020)	0.001
fibrinogen (mg/dl)	3.59 (3.03, 4.55)	4.1 (3.2, 5.6)	3.59 (3.03, 4.55)	(0.972,1.547)	0.044
**Echocardiography**					
LVEDD (mm)	48.25 (44.5, 53.64)	51.2 (48.1, 56.4)	48.25 (44.5, 53.64)	(1.012,1.128)	0.012
LVEF, %, (means ± SD)	41.78 ± 6.72	39.84 ± 10.05	41.78 ± 6.72	(0.886,0.981)	0.138
LV aneurysmn n (%)	19 (87.56)	15 (32.6)	19 (87.56)	(5.585,347.162)	0.001

The chest pain onset duration referred to the period of the occurrence of acute myocardial infarction to admission

*HTN* Hypertension, *DM* diabetes mellitus, *TIMI* thrombolysis in myocardial infarction, *DTBT* Door-to-balloon time, *WBC* White blood cell count, *MPV* Mean platelet volume, *NLR* Neutrophil /lymphocyte ratio, *PLR* Platelet/lymphocyte ratio, *LVEF* left ventricular ejection fraction, *LVEDD* left ventricular end-diastolic dimension, *LV* aneurysmn, left ventricular aneurysmn

*P* value represents the results of univariate analysis between LVT group and no LVT group

With respect to hematological parameters, the WBC, MPV and neutrophil and monocyte counts were not significantly different when the no-LVT group was compared to the LVT group (all *P* > 0.05). The number of lymphocytes in the LVT group was significantly lower than that in the no-LVT group (*p* = 0.001). The platelet count in patients with LVT was higher, but there was no significant difference between the two groups (*p* = 0.316). However, the PLR and NLR in patients with LVT were significantly higher than those in the no-LVT group (*P* = 0.001). Furthermore, the fibrinogen was significantly higher in the thrombus group (*P* = 0.044).

Echocardiographic parameters of patients, such as LVEF, ventricular aneurysm, and left ventricular diameter, were recorded and analyzed. No significant difference in LVEF was observed between the two groups (*P* > 0.05). The results demonstrated that both ventricular aneurysm and left ventricular diameter were significantly higher in the LVT group than in the no-LVT group (*P* < 0.05).

**Fig. 2:**
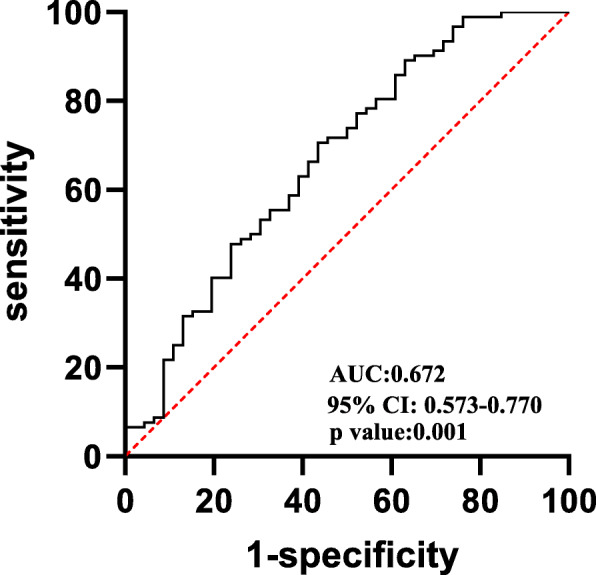
Receiver operating characteristic (ROC) curve analysis of platelet-to-lymphocyte ratio for predicting LVT formation. AUC: area under curve, CI: confidence interval

Further multivariate logistic regression analysis was used to determine the independent risks of LVT. After being adjusted for age, sex, and LVEF, the independent risk factors for LVT were an increased PLR (OR = 1.011, 95% CI: 1.004–1.018,*P* = 0.002), the presence of left ventricular aneurysm (OR = 46.350, 95% CI: 5.659–379.615, *P*<0.001) and an increased DTBT (OR = 1.005, 95% CI: 1.001–1.009, *P* = 0.012) (Table 2). Through the analysis of the ROC curve of the subjects (Fig. 2), a PLR > 118 (AUC: 0.672, 95% CI: 0.573–0.770, *p* = 0.001) was determined to have a sensitivity of 56.5% and a specificity of 70.7% for the prediction of thrombus in anterior STEMI with LV dysfunction after PCI. Moreover, in our study, 16 patients were diagnosed with the ventricular aneurysm, and 15 patients of them developed LVT. So, we conducted a sensitivity analysis removing the patients with LV aneurysm. In the analysis of the ROC curve of the subjects, the PLR > 121 (AUC: 0.676, 95% CI: 0.555–0.797, *p* = 0.006) had a 61.3% sensitivity and 69.4% specificity in predicting LVT in anterior STEMI with LV dysfunction after PCI. This result was consistent with our above finding in the whole subjects.

**Table 2:** Multivariate Logistic Regression Analysis for Assessment of Independent Predictors of LVT

Variable	OR	95%CI	***p*** Value	VIF
PLR	1.011	(1.004–1.018)	0.002	2.174
NLR	1.133	(0.966–1.328)	0.125	2.228
LV aneurysmn	46.350	(5.659–379.615)	<0.001	1.089
fibrinogen	1.248	(0.978–1.593)	0.074	1.328
DBTB	1.005	(1.001–1.009)	0.012	1.338
LVEDD (mm)	1.050	(0.990–1.114)	0.103	1.115

*PLR* Platelet/lymphocyte ratio, *NLR* Neutrophil /lymphocyte ratio, *LV* aneurysmn; left ventricular aneurysmn, *DBTB* Door-to-balloon time, *LVEDD* left ventricular end-diastolic dimension, *CI* confidence interval, *VIF* variance inflation factor

## Discussion

In this study, we observed that the PLR was significantly increased in patients with LVT. Increased PLR and the presence of left ventricular aneurysm were independent predictors of LVT formation in anterior STEMI complicated by LV dysfunction in patients treated with primary PCI. In the ROC curve analysis, the AUC for the PLR was 0.672, indicating that it is a promising index for the prediction of LVT formation in high-risk groups.

Although the reperfusion technique has been generalized during the acute infarction phase, the incidence of left ventricular thrombosis is still high [1]. Moreover, several studies [2, 7, 13, 14] reported that the sensitivity of transthoracic echocardiography (TTE) is as low as 20 to 25% when compared with cardiac magnetic resonance imaging with contrast-delayed enhancement (CMR-DE). The true incidence of LVT has been seriously underestimated in daily practice, most likely due to the limited use of CMR-DE. Furthermore, the prognostic effect of LVT was daunting. A recent meta-analysis [15] showed that LVT after acute myocardial infarction indicated a fourfold increased embolic risk and a twofold long-term mortality rate in the current era of PCI. The discovery of a more capable marker for the prediction of LV thrombus formation is a critical factor to avoid these terrible complications. Consistently known risk factors for LVT formation in STEMI include anterior location, lower LVEF, LV aneurysm, and delayed reperfusion [16–18]. Our study was precisely designed to identify the extra predictors of LVT in patients with the two main risk factors for LVT.

In the present analysis, LVT was more common in patients with longer DTBT Multivariate analysis showed that increased DTBT was an independent risk factor for the formation of LVT. Early data [19] showed that DTBT had a profound influence on the early patency of infarcted vessels. The two findings were likely related to delayed reperfusion, indicating that DTBT played a crucial role in STEMI patients. In contrast to our study, Rabbani et al. [20] found that DTBT was not an independent risk factor for LVT in anterior STEMI. The explanation might be that the subjects enrolled in our study were anterior STEMI with LV dysfunction patients, and delayed reperfusion played a more important role in such high-risk patients. In addition, compared to Rabbani’s study, the DTBT in our no-LVT group was relatively short, which might potentially reduce the influence of delayed reperfusion. Although thrombectomy was not recommended in routine application [21], De Rosa, S. et al. [22, 23] showed that thrombectomy had important positive effects on long-term clinical outcome in high-risk patients with AMI, and its effect became relevant in late presenters, suggesting that thrombectomy might be helpful in selected subpopulations at higher clinical risk. However, in our study, patients who were treated with thrombectomy showed no significant difference in the occurrence of LVT. The main explanation might be that 97.8% of the no-LVT group and 96.7% of the LVT group recovered to TIMI grade 3 during PCI. TIMI grade 3 flow before and after PCI is an independent predictor of 1-year survival [24]. Some previous studies [17, 25] have identified that the coactivation of coagulation and inflammation plays a central role in the formation of LVT. Shacham et al. [26] demonstrated that admission fibrinogen levels are independent predictors of early LVT formation in patients with first-ever anterior STEMI. This study was a survey of a general population and may not apply to our patients with systolic dysfunction. We also found a significantly higher level of fibrinogen in the LVT group. Regrettably, there was no statistical significance after multivariate analysis. Moreover, consistent with prior studies [27, 28], we also demonstrated an independent association between left ventricular aneurysm and LVT. Interestingly, sixteen patients were diagnosed with ventricular aneurysm, 93.75% (15/16) of whom had LVT. The formation of aneurysms caused by vortex reflux affects the normal deposition of blood, which will increase the incidence of LVT.

The PLR is an accessible, repeatable new biomarker of systemic inflammation and is related to the pathophysiological mechanism of inflammation and thrombosis. Platelets participate in the formation of blood clots and transmit mediators to develop and maintain an inflammatory response [29]. In contrast, lymphocytes are thought to control the inflammatory pathway. Higher platelet counts and lower lymphocyte counts are reported to be associated with adverse cardiovascular outcomes [30, 31]. The PLR can predict clinical outcomes in patients with systemic inflammation better than either the platelet count or the lymphocyte count alone. The interpretation of combined hematological indices may reveal patients at increased risk of thrombotic events. Insufficient standardization of laboratory measurements, inappropriate subject selection and preanalytical errors are the main limitations of the PLR [32]. A growing body of evidence now shows that an intriguing relationship does exist between high PLR and thrombotic diseases. Altintas et al. [33] found that a higher PLR is significantly associated with the presence of silent brain infarcts (SBIs) in patients with paroxysmal atrial fibrillation (PAF) (*P* = 0.001); increased PLR might be a factor that induces inflammatory processes on SBIs in patients with PAF even with low CHA2DS2–VASc scores. Kurtipek et al. [34] showed that in pulmonary thromboembolism (PTE) patients, using noninvasive ultrasonography combined with higher PLR in endothelial dysfunction diagnosis is effective. The study of Zuo et al. [35] proved that the PLR was a predictive marker of thrombogenesis in nonvalvular atrial fibrillation. Moreover, Gursoy et al. [36] performed a large-scale cohort study and stated that the PLR might be more specific than other inflammatory parameters for the indication of the presence of thrombosis in patients with prosthetic valve thrombosis (PVT). Multivariate analysis showed that an increased PLR was an independent predictor of thrombosis in patients with PVT. Similarly, in our study, the PLR was significantly higher in the LVT group, and lower lymphocyte levels probably contributed to this result because the platelet count between the two groups was not significantly different. The underlying mechanism [37] of low lymphocyte counts in patients with LVT has not been elucidated, but one explanation may be that cortisol inhibits the production of lymphocytes during acute stress. More specifically, it was found that [38–40] a decrease in lymphocyte count is a useful parameter describing atherosclerotic progression and adverse clinical outcomes in heart failure and acute coronary syndrome (ACS) patients. Hence, the PLR might be calculated as a simple, available parameter for individual risk stratification in high-risk patients to guide the choice of the best anticoagulant therapy strategy. It is expected that future studies on LVT will discover the potential mechanism of the prethrombotic effect of these new hematological parameters.

This study has some limitations. First, this was a single-center retrospective study involving a relatively limited number of patients and may not necessarily be representative of patient populations at other centers. Second, misdiagnosis or missed diagnosis may occur, but the likelihood of either when CMR-DE is used in the acute stage of myocardial infarction is decreased. Third, this study included only anterior STEMI patients post PCI with LV dysfunction. The selection of the patient population presents the possibility of selection bias. Moreover, our focus was on acute thrombosis (within 7 days of admission) formation, and we do not have information on additional late LVT formation.

## Conclusion

In patients with anterior STEMI and LV dysfunction, increased PLR and DTBT and the presence of LV aneurysm were associated with the presence of LVT and were independent predictors of LVT formation. In the future, the PLR may be valuable indicator to determine the occurrence of LVT in therapeutic practice. However, we must acknowledge that the results are hypothesis-generating rather than conclusive. Further studies are needed to clarify the mechanisms underlying the relationship between the PLR and LVT.

## Data Availability

The datasets used and/or analyzed during the current study are available from the corresponding author on reasonable request.

## Abbreviations

LVT: Left ventricular thrombus
PLR: Platelet/lymphocyte ratio
STEMI: ST-elevation myocardial infarction
PCI: Percutaneous coronary intervention
ROC: Receiver operator characteristic curve
AUC: Area under curve
DBTB: Door-to-balloon time
LVEF: Left ventricular ejection fraction
CMR-DE: Cardiac magnetic resonance imaging with contrast-delayed enhancement
